# Tauroursodeoxycholic acid in patients with amyotrophic lateral sclerosis: The TUDCA-ALS trial protocol

**DOI:** 10.3389/fneur.2022.1009113

**Published:** 2022-09-27

**Authors:** Alberto Albanese, Albert Christian Ludolph, Christopher J. McDermott, Philippe Corcia, Philip Van Damme, Leonard H. Van den Berg, Orla Hardiman, Gilberto Rinaldi, Nicola Vanacore, Brian Dickie, TUDCA-ALS Study Group, Paolo Tornese

**Affiliations:** ^1^Neurology Department, IRCCS Humanitas Research Hospital, Rozzano, Italy; ^2^Neurology Department, University of Ulm, Ulm, Germany; ^3^German Centre of Neurodegenerative Diseases, Site Ulm, Ulm, Germany; ^4^Department of Neuroscience, Sheffield Institute for Translational Neuroscience, University of Sheffield, Sheffield, United Kingdom; ^5^Centre de Référence Maladie Rare (CRMR) SLA et les autres maladies du neurone moteur (FILSLAN), Tours, France; ^6^CHU Bretonneau, Tours, France; ^7^Federation des CRMR-SLA Tours-Limoges, LITORALS, Tours, France; ^8^INSERM U1253, “iBrain,” Université François-Rabelais de Tours, Faculté de Médecine, Tours, France; ^9^Neurology Department, University Hospitals Leuven and Neuroscience Department, KU Leuven, Leuven, Belgium; ^10^Department of Neurology, UMC Utrecht Brain Center, University Medical Centre Utrecht, Utrecht, Netherlands; ^11^Academic Unit of Neurology, Trinity Biomedical Sciences Institute, Dublin, Ireland; ^12^Clinical Research Centre, Beaumont Hospital, Dublin, Ireland; ^13^Scientific Service, Bruschettini S.R.L, Genova, Italy; ^14^National Centre for Disease Prevention and Health Promotion, National Institute of Health, Rome, Italy; ^15^Motor Neurone Disease Association, Northampton, United Kingdom

**Keywords:** amyotrophic lateral sclerosis, therapy, clinical trial, phase III, bile acids

## Abstract

**Background:**

Amyotrophic lateral sclerosis (ALS) is a chronic neurodegenerative rare disease that affects motor neurons in the brain, brainstem, and spinal cord, resulting in progressive weakness and atrophy of voluntary skeletal muscles. Although much has been achieved in understanding the disease pathogenesis, treatment options are limited, and in Europe, riluzole is the only approved drug. Recently, some other drugs showed minor effects.

**Methods:**

The TUDCA-ALS trial is a phase III, multicenter, randomized, double-blind, placebo-controlled, parallel-group clinical trial. The study aims to enroll 320 patients in 25 centers across seven countries in Europe. Enrolled patients are randomized to one of two treatment arms: TUDCA or identical placebo by oral route. The study measures disease progression during the treatment period and compares it to natural progression during a no-treatment run-in phase. Clinical data and specific biomarkers are measured during the trial. The study is coordinated by a consortium composed of leading European ALS centers.

**Conclusion:**

This trial is aimed to determine whether TUDCA has a disease-modifying activity in ALS. Demonstration of TUDCA efficacy, combined with the validation of new biomarkers, could advance ALS patient care.

**Clinical trial registration:**

ClinicalTrials.gov, identifier: NCT03800524.

## Introduction

With an incidence of 0.3–2.5 cases per 100,000 people per year, amyotrophic lateral sclerosis (ALS) is a chronic neurodegenerative rare disease, affecting some 40,000 individuals in Europe, and causing around 11,000 deaths each year (1, 2). In 2015, ALS patients were estimated to be around 223,000 worldwide, a number expected to reach nearly 380,000 cases in 2040 (3). In ALS, disease progression is rapid and can be measured by survival or functional measures, but detecting clinically significant change still requires long treatment duration and large study populations (4). The pathogenic mechanisms proposed for ALS involve a plethora of alterations to the motor neuron microenvironment, including accumulation of protein aggregates, defective RNA processing, oxidative stress, glutamate excitotoxicity, axonal transport deficits, glial dysfunction, neuroinflammation, apoptosis, mitochondrial dysfunction, fragmentation of the Golgi apparatus, and metal imbalances (5). Although much has been achieved over the last two decades in understanding the disease complexity, currently there is no cure for ALS. The only approved treatment in Europe is riluzole, a glutamate release inhibitor, which was shown to slow the rate of progression and to slightly prolong survival (6). ALS has been the object of several controlled clinical trials focused on different molecules. Whether these trials have failed because of trial design and analysis or due to challenges in effectively targeting ALS pathophysiology remains uncertain (4, 7). There is a pressing need to find disease-modifying therapies that will slow ALS progression and enable patients to gain any possible length in survival.

A proof-of-concept phase IIb study showed that, in patients who received tauroursodeoxycholic acid (TUDCA, a potentially cytoprotective drug) in addition to riluzole for 54 weeks, the per-year decline rate in the revised-ALS functional rating scale was about seven points smaller compared to riluzole alone (8). This corresponds to a prolongation of median survival by 4–5 months. This indication of efficacy is further supported by the evidence that TUDCA has cytoprotective properties in animal models of different neurodegenerative diseases (5). Recently, another phase II trial on TUDCA combined with sodium phenylbutyrate showed a positive effect on disease progression, further supporting a possible role of TUDCA in reducing ALS progression (9). The TUDCA-ALS trial was developed to provide an answer on the clinical efficacy of TUDCA in ALS. This trial proposes an innovative design compared to earlier disease-modifying trials (4).

### Hydrophilic bile acids as potential disease modifiers

TUDCA is a hydrophilic bile acid that is normally produced endogenously in humans in the liver, by conjugation of taurine to ursodeoxycholic acid (UDCA). It is commonly used for the treatment of chronic cholestatic liver diseases and for gallstone (10). TUDCA is the active natural form of UDCA; it is orally bioavailable and blood–brain barrier permeable (5, 11). After oral administration, TUDCA is absorbed at the bowel level. The amount of bile salt that escapes hepatic uptake and reaches systemic circulation is reduced due to high efficiency of the hepatic *first-pass clearance* and intestinal absorption. TUDCA is taken up by the liver and excreted directly into bile, thus entering the enterohepatic circulation; in part, it is excreted with feces. Bile salts, including TUDCA, play a role in intestinal homeostasis by controlling the size and the composition of the intestinal microbiota (5, 12). Clinical studies performed on patients with different medical conditions over the last years unanimously report that the chronic administration of hydrophilic bile acids is safe and well-tolerated. It has been shown that TUDCA has neuroprotective effects in motor neuron–neuroblastoma hybrid cells expressing mutant superoxide dismutase 1 mutations A4V and G93A (13). Preclinical data also report an anti-apoptotic and neuroprotective role of TUDCA in models of different neurodegenerative diseases (5).

Preclinical demonstration of TUDCA neuroprotective activity is further supported by clinical evidence. Three pilot studies have been published on biliary acids in patients with ALS. A phase II study administered UDCA to 18 ALS patients, randomly assigned to receive 15, 30, or 50 mg/kg UDCA daily (11). The drug was well-tolerated by all subjects at all doses. UDCA reached meaningful serum levels after oral intake and was also detected in the CSF of treated patients. An oral soluble UDCA formulation (3.5 g/140 mL/day) was tested for 3 months on 64 ALS patients in a phase II crossover trial (14). The study reported a possible beneficial effect of UDCA on functional decline in ALS. A subsequent phase II double-blind placebo-controlled study evaluated the safety and efficacy of 34 ALS patients taking riluzole and randomized to add placebo or TUDCA (1 g twice daily for 54 weeks) (8). The dose was chosen considering the high hepatic uptake of TUDCA that limits the amount reaching systemic circulation and crossing the blood–brain barrier, and on non-clinical safety studies reporting that TUDCA has low toxicity. The study showed that TUDCA was well-tolerated at the dose used in the clinical trial. Mild diarrhea occurred in two patients treated with TUDCA and in two treated with placebo; anorexia was reported in a placebo-treated patient. Treatment with TUDCA for 1 year at a dose of 2 g daily was associated with a potentially slower deterioration of function in ALS patients. The primary efficacy measure of that study was met, because the proportion of responders was higher in the TUDCA than in the placebo arms (87 vs. 43%, *P* = 0.021) (8). According to this study design, responding patients were those with a ≥15% reduction in the ALSFRS-R slope decline at study end. Furthermore, this 1-year study showed that treatment with TUDCA was associated with a slower deterioration of function in ALS patients (secondary efficacy measure). In the patients who received TUDCA and riluzole, the ALS functional rating scale-revised (ALSFRS-R) per-year decline rate was about seven points smaller than with riluzole only. On the 0–48 ALSFRS-R score, this corresponds to a prolongation of median survival by 4–5 months. In summary, the pilot studies showed that TUDCA is safe and may be beneficial in ALS.

### Rationale

The promising results obtained from phase II clinical studies strongly encouraged to establish a large multicenter phase III trial, to confirm and further measure the efficacy of TUDCA as a disease modifier in ALS.

The TUDCA-ALS consortium was therefore set up. This is composed of European centers with established experience in ALS and deep-rooted services for ALS. The consortium received funding from the European Commission to conduct a well-designed clinical trial under the Horizon 2020 call SC1-PM-08-2017 “New therapies for rare diseases.” The TUDCA-ALS incorporates the design and experience of the earlier phase II TUDCA study (8) with strengthened endpoints and the addition of innovative biomarker analysis. Responder analysis provides an innovative clinical design for ALS studies, allowing to overcome several methodological difficulties observed in the classical parallel-group design (4). Responder analysis assesses individual ALSFRS-R decline and censors each patient according to variations of post- vs. pre-treatment disease progression trajectories. In an earlier phase II study, responding patients were defined as those showing a mitigation of the ALSFRS-R progression slope by at least 15% in the treatment period compared to the run-in period (8). In this trial, the threshold for censoring patients as treatment responsive was increased to 20%, based on suggestions received by the EMA Committee for Orphan Medicinal Products (COMP). Furthermore, a separate *post-hoc* analysis with a 25% threshold has also been planned. These changes took into account a survey among experienced ALS clinical investigators reporting that 25% or higher changes in the ALSFRS-R slope were considered clinically meaningful (15).

The number of responding patients is the primary outcome measure of the efficacy in the TUDCA-ALS study. The study also includes endpoints based on biomarkers related to disease progression and to cytoprotective activity. Neurofilaments are an accepted neuron-specific structural biomarker of motor axons (16). Cerebrospinal fluid (CSF) levels of phosphorylated neurofilament heavy chain (pNfH) are increased in patients with ALS, and NF light chain (NFL) levels correlate with ALS progression rate (17). The TUDCA-ALS protocol also includes matrix metalloproteinase-9 (MMP-9) as an additional biomarker signature for ALS. MMP-9 is elevated in tissues and biofluids of ALS patients (18). Elevated concentrations of MMP-9 in serum of ALS patients were associated with muscle denervation and extensive neuroaxonal degeneration causing motor neuron loss (19). Finally, plasma creatinine was recently reported by a robust biomarker of disease progression in ALS, suggesting a potential usage as an endpoint in clinical trials (20).

### Objectives

The objective of the TUDCA-ALS trial is to assess the efficacy of TUDCA in ALS sufferers by censoring patients with a disease-modifying effect during the experimental treatment. Our hypothesis is that the number of patients with a more benign disease progression trajectory is expected to be higher in the treated arm.

## Materials and methods

### Study design and setting

This is a phase III, multicenter, randomized, double-blind, placebo-controlled, parallel-group study. Enrolled participants are randomized to one of two treatment arms: TUDCA or identical placebo by oral route. The use of placebo is justified by the consideration that at present, there is no cure for ALS. The trial medication is administered as add-on to an optional standard therapy with riluzole, a licensed drug for ALS in Europe.

**Figure 1:**
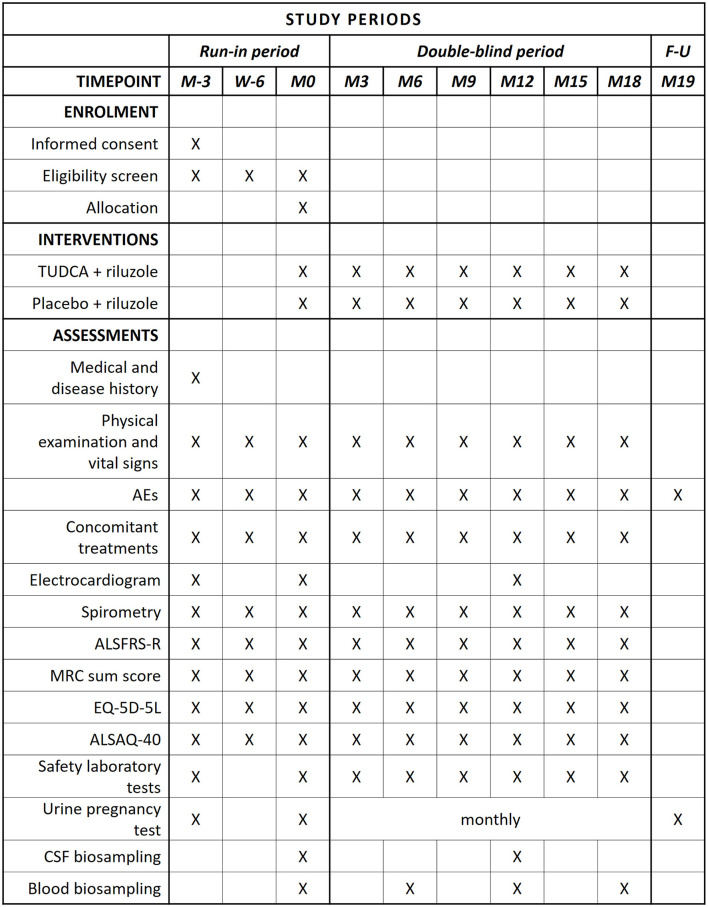
SPIRIT schedule of enrollment, interventions, and assessments. In compliance with current marketing authorization for TUDCA, the following liver function parameters are monitored: AST, ALT, GGT, and bilirubin. Considering that there is yet insufficient information on possible teratogenic effects, a urine pregnancy test is performed monthly (from M0 to M18) and 30 days (1 month) after the last IMP dose.

**Figure 2:**
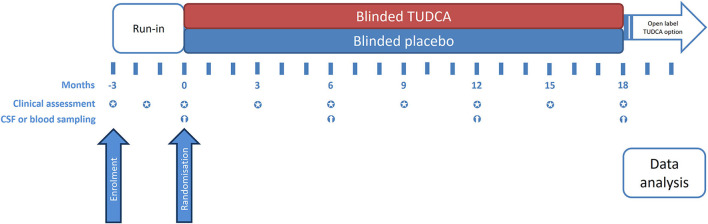
Timeline of the study and visit schedule.

The present study implements a run-in design to collect observations on natural disease progression before administering the investigational medicinal product (IMP) (4). The study timeline, procedures, and scheduled visits are detailed in Figures 1, 2. Briefly, during the 3-month run-in period, the patients receive riluzole at the stable dose of 50 mg twice daily (100 mg daily). Three assessments at 6-week intervals are performed during this period, to measure the disease progression rate before starting study treatment. Upon completion of the run-in period, the eligible patients are randomized to one of the two treatment arms. The IMP is administered orally at the dose of 1 g twice daily for 18 months in addition to riluzole. Clinical assessments during the double-blind phase are performed every 3 months to measure disease progression during treatment with the IMP. Progression trajectories under the experimental treatment are compared to pre-treatment natural progression (measured during the run-in period).

This study design is in keeping with a consensus on clinical trial design in ALS (21) and has been reviewed in detail (4). The categorical design has the advantage to accommodate for individual variabilities in progression trajectories and for potential heterogeneities in response to medications. For patient censoring, responding patients are defined as having a ≥20% reduction in the ALSFRS-R slope decline during the investigational treatment period, compared to run-in (15).

The trial is completed when the last study visit of the last enrolled patient is carried out. After the trial end, the patients are offered the possibility to receive open-label active treatment, remaining blind to experimental treatment until the study unblinding.

The setting is in the outpatient clinic or day service. The study is conducted in 25 clinical trial units located in Belgium, France, Germany, Ireland, Italy, Netherlands, and United Kingdom.

### Ethics approval

The TUDCA-ALS consortium implements this study in full respect of the legal and ethical European requirements and codes of practices on the conduct of clinical trials. All procedures involving human beings conform to the Declaration of Helsinki and to Good Clinical Practices (GCPs) (22). Written informed consent is obtained from the enrolled study subjects at the initial screening visit. A contract research organization (CRO) controls the ethical standards of the planned work and guarantees that all the personnel involved in the study is adequately trained on ethical and safety requirements.

The trial protocol received EudraCT number 2018-002722-22 and was registered on ClinicalTrials.gov as NCT03800524. The full protocol, the informed consent forms (ICFs), and all supporting documents are approved by all the involved ethics committees and by national regulatory authorities. The original protocol was amended on 23 July 2021 to include changes concerning inclusion and exclusion criteria, sample size, the open-label extension, and the implementation of mitigation actions related to COVID-19 outbreak (described in Table 1). The study starts only after the full sequence of approvals is completed.

**Table 1:** COVID-19 related mitigation actions.

The COVID-19 outbreak occurred while the trial was running at month 26. The following actions have been implemented to pursue completion of the trial.
• In case of national or local restrictions that prevent the subject from attending the study site, the visits from week 6 onward can be performed at distance, using appropriate audio-visual connections (including telephone contacts) with the patient at home. The telemedicine-based strategy can be applied exceptionally also to the screening visit.
• The patients are required to perform at a minimum the safety laboratory tests scheduled at M-3, M0, M3, and at least once every 6 months thereafter; spirometry can be avoided for all visits, except for the screening visit (M-3).
• Safety laboratory tests and spirometry can be performed in a different center, according to the patient's best convenience; women of childbearing potential are required to take the urine pregnancy tests foreseen by the protocol at their convenience.
• The results of these tests have to be collected *via* telephone, e-mail, or other informatics means by delegated personnel only. The results can also be collected by the investigator at the first in-person visit at the clinical center and are stored in the patient's folder.
• All events related to COVID-19 must be reported as adverse events or serious adverse events, as appropriate, and recorded in the eCRF.

### Eligibility

Enrollment is open to patients of either gender, aged from 18 to 80 years inclusive. Patients already taking riluzole are eligible candidates. All eligibility criteria (Tables 2, 3) are to be fulfilled prior to randomization visit (month 0).

**Table 2:** Inclusion criteria.

1. Probable laboratory-supported, probable, or definite ALS, as defined by El Escorial Revised ALS diagnostic criteria at screening visit (month−3) (23)
2. Disease duration ≤ 18 months at screening visit (month−3)
3. Able to perform reproducible pulmonary function tests at screening visit (month−3)
4. Forced vital capacity or slow vital capacity ≥70% of normal at screening visit (month−3)
5. Stable on riluzole treatment for 3 months in the lead-in period
6. Signed informed consent at screening visit (month−3)

**Table 3:** Exclusion criteria.

1. Treatment with edaravone or other unaccepted concomitant therapy [e.g., substances inhibiting the intestinal absorption of biliary acids, such as cholestyramine, colestipol; antacids containing aluminum hydroxide and/or smectites (aluminum oxide); estrogens and drugs acting by lowering plasmatic cholesterol, such as clofibrate; drugs increasing biliary clearance of cholesterol (estrogens, hormonal contraceptives, some hypolipaemizing agents); hepatolesive drugs]
2. Other causes of neuromuscular weakness
3. Presence of other neurodegenerative diseases
4. Clinical evidence of cognitive impairment, dementia or psychiatric illness
5. Severe cardiac or pulmonary disease
6. Other diseases precluding functional assessments
7. Other life-threatening diseases
8. Any use of non-invasive ventilation (e.g., continuous positive airway pressure, non-invasive bi-level positive airway pressure or non-invasive volume ventilation) for any portion of the day, or mechanical ventilation *via* tracheostomy, or on any form of oxygen supplementation
9. Gastrointestinal disorder that is likely to impair absorption of study drug from the gastrointestinal tract
10. Has taken any investigational study drug within 30 days or five half-lives of the prior agent, whichever is longer, prior to dosing
11. Any clinically significant laboratory abnormality
12. Other concurrent investigational medications
13. Active peptic ulcer
14. Previous surgery or infections of small intestine
15. Patients unable to easily swallow the treatment pills
16. Acute inflammation of the gallbladder or bile ducts
17. Occurrence of frequent biliary colic, biliary infections, severe pancreatic abnormalities
18. Bile duct obstruction, calcified X-ray opaque gallstones and reduced mobility of the gallbladder
19. Subjects who weigh 88 lbs (40 kg) or less
20. Aspartate aminotransferase or alanine aminotransferase concentrations more than 3 times the upper limit of normal
21. Creatinine clearance 50 ml/min or less
22. Any clinically significant neurological, hematological, autoimmune, endocrine, cardiovascular, neoplastic, renal, gastrointestinal, or other disorder that, in the Investigator's opinion, could interfere with the subject's participation in the study, place the subject at increased risk, or confound interpretation of study results
23. Consideration by the investigator, for any reason, that the subject is an unsuitable candidate to receive TUDCA or that the subject is unable or unlikely to comply with the dosing schedule or study evaluations
24. The patient of reproductive potential is sexually active and is not willing to use highly effective contraception during the study and up to 90 days after the day of last dose
25. The patient is pregnant or breast feeding

### Screening and consent

The patients are enrolled at each participating center. Before entering the study, the patients are informed about the purposes of the trial, possible benefits, and potential personal reasonable risk or discomfort, the expected duration of their participation, the procedures and laboratory tests they will undergo, as well as the name and contact details of the investigators responsible for conducting the trial. Patients are free to choose whether to take part. Their decision is voluntary, and they should be competent to understand what is involved: Persons under guardianship or considered unable are excluded. The investigators also inform the patients that they can leave the study at any time and for any reason without giving an explanation and that this discontinuation would not in any case deteriorate the relationship with the physician or the possibility to receive alternative therapies. Before recruitment, each patient receives a copy of the informed consent form together with all needed clarifications. Sufficient time is given to enable the patient to take a decision on whether to participate to the study. If the patient agrees to participate, he/she personally signs and dates the form. If a study participant is unable to write, oral consent in the presence of at least one impartial witness is provided.

### Run-in observation

Run-in provides to establish baseline measurements for comparison after the intervention has been applied. In this study, the run-in period lasts for 3 months, during which three assessments are performed at the following times: 3 months before the start of the double-blind phase (M-3), 6 weeks before the start of the double-blind phase (W-6), and at the start of the double-blind phase (M0).

At the end of the screening visit (M-3), after signing the ICF, patients enter the run-in period and are assigned a univocal patient number, consisting of a predefined four-digit center-specific code, followed by a sequential two-digit consecutive patient enrollment number. This pseudonymized code remains constant throughout the study and is used to identify patients in the electronic case report form (eCRF). Disease progression during the run-in period is not a criterion for excluding patients and serves only to draw the baseline progression trajectory. However, all exclusion criteria are to be fulfilled throughout the run-in period and up to the randomization visit (month 0).

### Randomization

Before the trial start, the statisticians at the Italian Institute of Health (IIH) generated randomization lists to allocate patients to TUDCA or placebo arms. Allocation to treatment arms is performed on a 1:1 ratio. Randomization is stratified by country. Within each stratum/country, the sequence of treatments is randomly permuted in balanced sequences of four. This short sequence length and the lack of restriction in the number of patients enrolled in each stratum guarantees factor balancing during the recruitment phase. The unblinded randomization lists are sent to identified delegated personnel at the IMP packaging facility. These lists are used to label IMP boxes with the appropriate treatment code. The randomization lists are stored at IIH and at the IMP packaging facility; all other personnel involved in the partnership or in the trial remains blind to treatment assignment throughout the entire duration of the project.

At month 0, upon completion of the run-in period, patients are randomized to one of two treatment arms, by means of a computerized central randomization system *via* the eCRF. IIH provided the blinded randomization lists to eCRF data managers prior to the trial start; they are responsible for implementing the randomization function through eCRF platform that randomly couples each patient to a treatment code. The patient then receives IMP boxes labeled with the same treatment code assigned by the randomization module.

### Intervention

The IMP can be either active treatment (TUDCA, 250 mg capsules) or matching placebo. TUDCA is licensed in Italy and in some non-European countries (e.g., Turkey, China) under the tradename Tudcabil^®^ for the treatment of alterations of the bile production by the liver; its marketing authorization holder is Bruschettini S.r.l. TUDCA has been granted orphan drug designation by the European Medicines Agency on 27 February 2017 (EU designation EU/3/17/1844). The IMP is provided in blisters of 10 capsules each, identical for TUDCA and placebo. The IMP is distributed by an authorized facility for packaging and distribution of drug and placebo treatments for clinical trials.

The trial procedures are shown in Figure 1.

### Outcomes

The primary outcome is the proportion of responding patients in each treatment arm. Responding patients are defined as those showing a reduction in the ALSFRS-R slope decline by at least 20% when comparing the 18-month trial to the run-in period. For each randomized patient, slope coefficients of ALSFRS-R are calculated by linear regression models separately during the run-in period and during the 18 months of treatment.

The secondary outcomes of the study include the following:

1. Survival time measured by death or respiratory insufficiency, defined as tracheostomy or the use of non-invasive ventilation for ≥22 h per day for ≥10 consecutive days.
2. Difference in change from baseline in disease progression and functional impairment between TUDCA and placebo over 18 months, as measured by ALSFRS-R, ALSAQ-40, FVC, EQ-5D-5L, and MRC sum score.
3. Long-term safety and tolerability of TUDCA treatment in patients with ALS for up to 18 months, as assessed through adverse reaction, concomitant treatment, physical examination, vital signs, and routine hematology and biochemistry analyses.
4. Effect of TUDCA on biomarkers of disease progression, such as CSF and serum neurofilament levels, serum MMP-9 expression, and plasma creatinine levels, evaluated on biological samples collected at the scheduled visits.

Collection of CSF samples was not a mandatory feature, considering different practices within Europe on performing lumbar puncture in ALS patients.

### Statistical plan

The study is powered to independently assess a potential benefit of TUDCA compared with placebo on the progression of ALSFRS-R total scores. In the original planning, the minimum detectable effect was calculated by setting power to 80% and type I error at 5%. Based on data collected by the phase II trial (8), it was assumed a response rate of 10% in the placebo arm and of 20% in the active arm. A sample size of 400 patients was then considered appropriate to detect a treatment effect of 10%, corresponding to a risk ratio (RR) of 2.00. The COVID-19 outbreak occurred soon after the trial start and significantly slowed trial activities in all countries, particularly patient enrollment. The enrollment phase was then extended by 18 months, and a scenario considering a reduced sample size was developed. It is reckoned that a size of 320 participants could be sufficient to detect a statistically significant 11% treatment effect, assuming a response rate of 21% in the active arm and a 2.14 RR.

The primary analysis is carried out on the primary endpoint in the intention-to-treat (ITT) population, defined as all the randomized patients who received at least one dose of treatment. Slope coefficients for ALSFRS-R decline are calculated by a linear regression model during the run-in period and during the 18-month treatment period. For each patient, all the available ALSFRS-R scores are included in the linear regression model for the estimation of monthly decline. Responding patients are defined as those with a ≥20% reduction in ALSFRS-R slope decline comparing the 18-month treatment period with run-in. In the secondary analysis, all efficacy and safety endpoints are compared between the two randomization arms at study end on the ITT population. *Post-hoc* analyses will be performed based on clinical (e.g., bulbar vs. limb onset) or biological stratification. For all statistical analyses, the level of statistical significance is kept at 0.05 with two-sided *p*-values.

Statistical analysis is performed upon completion of the study, using patient group assignments as group A vs. B (triple-blind scheme). When all analyses are performed and the final report is drafted, treatment assignment to groups will be unblinded.

### Safety

TUDCA is generally well-tolerated. Safety data on TUDCA and other hydrophilic bile acids have been recently reviewed (5). Adverse reactions related to the use of TUDCA in the currently approved indications include cases of soft feces and diarrhea. Nausea, vomiting, or loose stools are rare occurrences, whereas hives or gallstone calcifications have been reported very rarely. Previous clinical trials in ALS patients reported soft feces or transient diarrhea after the initial doses (8, 9, 24). If there is diarrhea after starting the IMP, a dose reduction to 1 g daily (0.5 g b.i.d.) is permitted for up to 2 weeks to allow for adaptation.

All serious adverse reactions occurring during clinical studies are reported to the sponsor or its delegate by the investigational staff within 24 h of their knowledge of the event. The cause of death, when the event is associated with the investigational agent, is a serious adverse reaction.

### Management and monitoring

**Figure 3:**
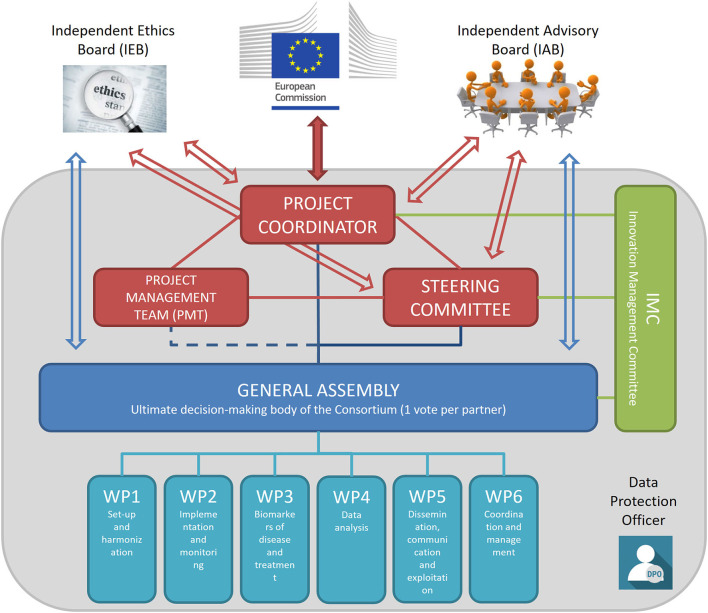
TUDCA-ALS management structure. Coordinating bodies are shown in red. The Project Coordinator is responsible for the core management tasks and the overall progress of the project; the Project Management Team is in charge of the project management and administration, in close collaboration with the Project Coordinator; the Steering Committee implements decisions taken by the General Assembly and reviews the project results. The General Assembly (shown in blue) is the decision-making body of the project overseeing the six work packages. The Innovation Management Committee (shown in green) monitors the principles for Intellectual Property rights and the dissemination and exploitation of project results. The Independent Advisory Board provides external advice on the conduct of the project, to bring maximum impact. The Independent Ethics Board provides guidance on ethical issues. The Data Protection Officer assures that trial activities conform to current EU and national legislation.

The management structure of the consortium and the trial includes several bodies with different roles and duties that interact transversally to assure that all activities pertaining to the trial are conducted in full compliance with GCP guidance and national regulations. As detailed in Figure 3, the main components of the management structure are the coordinating bodies, including the Project Coordinator, the Steering Committee, the Project Management Team, and the General Assembly that is the main decision-making body. In addition, the Independent Advisory Board, the Independent Ethics Board, and the Data Protection Officer provide external oversight and advice on the conduct of the study.

The Independent Ethics Board-Data Safety Monitoring Board (IEB-DSMB) provides independent guidance on ethics and safety issues that could arise from the project. The IEB-DSMB is composed of an Ethics Expert, for advice on ethics, and a Clinical Expert, for clinical and safety advice. The two experts are independent from the TUDCA-ALS consortium, do not act as investigators or sub-investigators for this study, have not participated in the design of the clinical trial protocol, and do not belong to any partner institution. The Motor Neurone Disease Association (MNDA), the largest ALS patient charity in Europe and partner of the TUDCA-ALS consortium, is an *ex officio* non-voting member of the IEB-DSMB. The IEB-DSMB has a consultative role; it can make recommendations to the appropriate executive committees of the TUDCA-ALS consortium during the project, but does not have the faculty to request modifications to the study design and objectives, or to take stopping decisions about the trial. The IEB-DSMB Clinical Expert has the role to alert the Steering Committee whenever in his view serious safety concerns need to be addressed by the consortium. The Clinical Expert may request unblinding from treatment allocation, should serious safety concerns be suspected. In this case, the Clinical Expert alone is unblinded, whereas all the other personnel remains blinded. The IEB-DSMB charter defines the board composition, its duties and responsibilities, as well as its relationship with other consortium bodies.

Personal data are processed only for the purpose of the clinical research project. All records identifying the patients are kept confidential and, to the extent permitted by the applicable laws, not made publicly available. Electronic data are stored in the eCRF for the entire duration of the study, until transferred to IIH for statistical analysis. Biological samples are collected at the centers during scheduled visits and then processed locally. Biological samples are transferred to the centralized laboratories for the assessment of biomarkers. The results of the analyses on biomarkers are then transferred to IIH, where they are analyzed statistically in connection with clinical outcomes.

**Figure 4:**
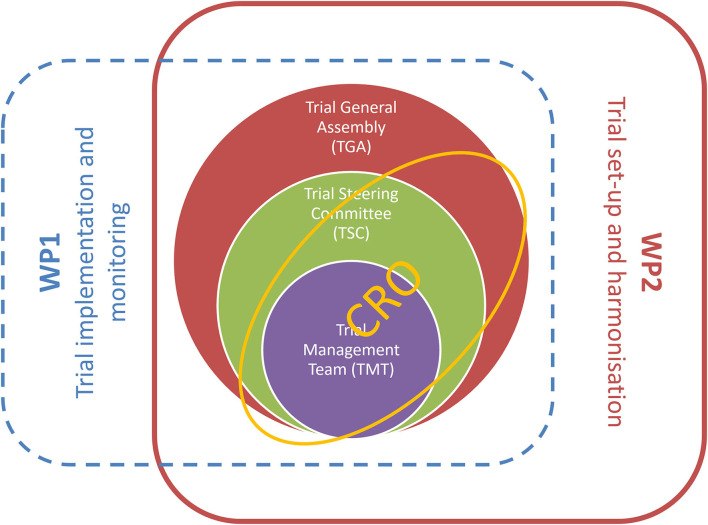
Trial management structure is nestled into activities of workpackages 1 and 2 of the TUDCA-ALS consortium. Workpackage 1 is responsible for trial implementation and monitoring, and workpackage 2 is dedicated to trial setup and harmonization. These core activities are implemented through intertwined interactions between the Trial Management Team, the Trial General Assembly, the Trial Steering Committee, and the contract research organization.

The trial management structure is nested into the management structure of the TUDCA-ALS consortium. The operations related to trial setup, implementation, and monitoring are realized through the intertwined actions of three bodies: the Trial Management Team, the Trial General Assembly, and the contract research organization (CRO), as represented in Figure 4. A CRO monitors the progress of the trial, ensuring that it is conducted, recorded, and reported in accordance with the study protocol, standard operating procedures, good clinical practice, and relevant European and national regulatory requirements of all the countries involved. Before the study, the CRO collected the essential documents needed for regulatory and ethics approvals. The CRO organizes site initiation visits for all centers, to check that the complete trial documentation is available on site and that all protocol procedures are well-understood by the investigating staff.

### Trial status

The most recent protocol version is version 2.0, which was approved and signed on 23 July 2021. The sites started recruiting patients in February 2019 across the seven involved countries. Enrollment was completed in December 2021, and the last patient is expected to complete the trial in October 2023. A manuscript detailing the results of the trial is expected to be submitted for the publication by the end of 2023.

## Discussion

### Dissemination policy

Sharing TUDCA-ALS data is an objective of the consortium. The MNDA aids in promotion and diffusion of trial outcomes to the general public and to key stakeholders, including the patients. Each partner guarantees open access to all peer-reviewed scientific publications relating to TUDCA-ALS results. Once the dataset has been analyzed, a complete, cleaned, anonymized copy of data used in conducting the final analyses can be made available to qualified, designated persons from other academic institutions or private companies on request, *via* a data repository accessible through Internet by registered users. Patient anonymity and legal compliance are assured throughout all the steps of data transfer. After the trial, biological samples collected during the trial are kept in the Ulm NeuroBioBank and used for further exploratory analysis or shared with other organizations.

### Clinical perspectives

The TUDCA-ALS study is the largest clinical trial ever conducted to evaluate the efficacy of TUDCA as add-on treatment in people with ALS, who suffer from one of the most devastating neurodegenerative disorders. Despite the recent insights into the molecular mechanism of ALS, the therapeutic options for ALS patients have not improved in the last years. Drug candidates that ameliorate symptoms are available, but they provide no benefits in terms of survival or delayed onset. There is hope to find innovative treatments that can be added to current medications to slow-down the disease process, particularly in the early stages. Considering the encouraging results of two phase II clinical trials testing the effects of TUDCA alone or in combination (8, 9), the TUDCA-ALS project promises to provide innovative answers on the clinical management of ALS patients.

## Ethics statement

The study was reviewed and approved by all the involved national and local Ethical Committees (the full list is available as a Supplementary File). The patients/participants provided their written informed consent to participate in this study.

## Author contributions

AA conceived, wrote, and finalized the manuscript. AL, CJM, PC, PV, LV, OH, GR, NV, and BD reviewed and edited the manuscript. All authors contributed to the article and approved the submitted version.

## Collaborators of the TUDCA-ALS study group

Paolo Tornese, Antoniangela Cocco, Maria Lo Giudice, Department of Neurology, IRCCS Humanitas Research Hospital, Rozzano (Italy); Michela Matteoli, Eliana Lauranzano, Maria Luisa Malosio, Chiara Adriana Elia, Laboratory of Pharmacology and Brain Pathology, IRCCS Humanitas Research Hospital, Rozzano (Italy) and Institute of Neuroscience, National Research Council, Rozzano (Italy); Flavia Lombardo, Flavia Mayer, National Center for Disease Prevention and Health Promotion, National Institute of Health Rome (Italy). Maria Puopolo, Department of Neuroscience, National Institute of Health, Rome (Italy). Stefania Spila Alegiani, National Center for Drug Research and Evaluation, National Institute of Health, Rome (Italy); Adriano Chiò, Umberto Manera, Cristina Moglia, Andrea Calvo, Paolina Salamone, Giuseppe Fuda “Rita Levi Montalcini” Department of Neuroscience, University of Turin (Italy); Carlo Colosimo, Cristina Spera, Prabha Cristina Ranchicchio, Department of Neurology, Azienda Ospedaliera Santa Maria, Terni (Italy). Giuseppe Stipa, Domenico Frondizi, Department of Neurophysiology, Azienda Ospedaliera Santa Maria, Terni (Italy); Christian Lunetta, Valeria Sansone, Claudia Tarlarini, Francesca Gerardi, Centro Clinico NEMO Fondazione Serena ONLUS, Milan (Italy); Vincenzo Silani, Alberto Doretti, Eleonora Colombo, Gianluca Demirtzidis, Department of Neurology, IRCCS Istituto Auxologico Italiano, Milan (Italy) and Department of Pathophysiology and Transplantation, “Dino Ferrari” Center, Università degli Studi di Milano, Milan (Italy); Gioacchino Tedeschi, Francesca Trojsi, Carla Passaniti, Department of Advanced Medical and Surgical Sciences, First Division of Neurology, Università degli Studi della Campania “Luigi Vanvitelli,” Naples (Italy); Stefania Ballestrero, Bruschettini S.r.l, Genova (Italy); Johannes Dorst, Ulrike Weiland, Andrea Fromm, Maximilian Wiesenfarth, Katharina Kandler, Simon Witzel, Markus Otto, Joachim Schuster, Department of Neurology, University of Ulm, Ulm (Germany); Thomas Meyer, André Maier, Dagmar Kettemann, Charité — Universitätsmedizin Berlin, Corporate Member of Freie Universität Berlin and Humboldt-Universität Zu Berlin and Berlin Institute of Health, Outpatient Center for ALS and Other Motor Neuron Diseases, Berlin (Germany); Susanne Petri, Lars Müschen, Camilla Wohnrade, Anastasia Sarikidi, Alma Osmanovic, Department of Neurology, Hannover Medical School, Hannover (Germany); Julian Grosskreutz, Annekathrin Rödiger, Robert Steinbach, Benjamin Ilse, Uta Smesny, Department of Neurology, University Hospital Jena, Jena (Germany); Robert Untucht, René Günther, Maximilian Vidovic, University Hospital Carl Gustav Carus, Technische Universität Dresden, Dresden (Germany); Pamela Shaw, Alexis Collins, Helen Wollff, Theresa Walsh, Lee Tuddenham, Mbombe Kazoka, David White, Stacy Young, Benjamin Thompson, Daniel Madarshahian, Department of Neuroscience, Sheffield Institute for Translational Neuroscience, University of Sheffield, Sheffield (UK); Suresh K. Chhetri, Lancashire Teaching Hospitals NHS Foundation Trust, Preston (UK); Amina Chaouch, Manchester Centre for Clinical Neuroscience, Salford Royal NHS Foundation Trust, Northern Care Alliance M6 8HD Salford (UK); Carolyn A. Young, Heike Arndt, The Walton Centre NHS Foundation Trust, Liverpool (UK); C Oliver Hanemann, Peninsula Medical School, University of Plymouth, Plymouth (UK); Thomas Lambert, Royal Stoke University Hospital, Stoke on Trent (UK); Stephane Beltran, Centre Constitutif de référence SLA, CHU Bretonneau, 2, boulevard Tonnelle, 37044 Tours cedex 1, France; Fédération des Centres SLA De Tours et Limoges, LITORALS, Tours (France); Philippe Couratier, Hôpital Dupuytren - Service de Neurologie 2 - Cedex Clinique du Motoneurone - CRMR SLA, Limoges (France); Florence Esselin, William Camu, Elisa De La Cruz, Explorations neurologiques et centre SLA, Univ Montpellier, CHU Gui de Chauliac, INM, INSERM, Montpellier, France; Gwendal Lemasson, Groupe Hospitalier Pelegrin - Tripode - Pôle Neurosciences cliniques - Service de Neurologie Tripode, Bordeaux (France); Pegah Masrori, Neurology Department, University Hospitals Leuven and Neuroscience Department, KU Leuven, Leuven, Belgium; Sinead Maguire, Liz Fogarty, Toyosi Atoyebi, Niamh Ní Obáin, Academic Unit of Neurology, Trinity Biomedical Sciences Institute, Dublin; Clinical Research Centre, Beaumont Hospital, Dublin (Ireland).

## Funding

This is an investigator-initiated study funded by the European Union's Horizon 2020 research and innovation program (grant agreement 755094).

## Conflict of interest

Author AL declares participation in Advisory Boards of Roche Pharma AG, Biogen, Alector, and Amylyx. Author CJM reports consultancy with Biogen, Amylyx, and Cytokinetics. Author PV declares participation in Advisory Board meetings for Biogen, UCB, argenx, Cytokinetics, Ferrer, Muna Therapeutics, Augustine Therapeutics, Alector, Alexion, QurAlis, and VectorY (paid to institution) and grant from CSL Behring (E. von Behring Chair for Neuromuscular and Neurodegenerative Disorders; paid to institution). Author OH declares participation in Advisory Boards for Accelsiors, Biogen Idec, Cytokinetics, NeuroSense Therapeutics, Neuropath Therapeutics, Novartis, Orion, Denali, and Wave Pharmaceuticals; role as Principal Investigator on the PRECISION ALS Project; Academic/Industry Collaboration funded by Science Foundation Ireland; Research collaboration with Biogen, Cytokinetics, and Ionis in delivering the IMPACT ALS survey and with Cytokinetics in delivering the REVEALS study of respiratory decline in ALS; Editor-in-Chief of ALS and the Frontotemporal Degeneration; Editorial board member of the Journal of Neurology, Neurosurgery, and Psychiatry. Author GR declares that Bruschettini S.R.L is the pharmaceutical company providing the investigational medicinal product and industrial partner of the project, in which he is an employee as Scientific Director. The remaining authors declare that the research was conducted in the absence of any commercial or financial relationships that could be construed as a potential conflict of interest.

## Publisher's note

All claims expressed in this article are solely those of the authors and do not necessarily represent those of their affiliated organizations, or those of the publisher, the editors and the reviewers. Any product that may be evaluated in this article, or claim that may be made by its manufacturer, is not guaranteed or endorsed by the publisher.

## Abbreviations

ALS: amyotrophic lateral sclerosis
ALSAQ40: ALS Assessment Questionnaire-40
ALSFRS-R: ALS functional rating scale-revised
CRO: contract research organization
CSF: cerebrospinal fluid
eCRF: electronic case report form
EQ-5D-5L: EuroQol 5-Dimension-5L
FVC: forced vital capacity
GCP: good clinical practice
IMP: investigational medicinal product
IIH: Italian Institute of Health
ITT: intention to treat
MMP-9: matrix metalloproteinase-9
MRC: Medical Research Council
NFL: neurofilament light chain
pNFH: neurofilament heavy chain
SOPs: standard operating procedures
TUDCA: tauroursodeoxycholic acid
UDCA: ursodeoxycholic acid.
