# Supplementary material for: Tauroursodeoxycholic acid in patients with amyotrophic lateral sclerosis: The TUDCA-ALS trial protocol
Source: Front Neurol. 2022 Sep 27;13:1009113. doi: 10.3389/fneur.2022.1009113 (PMC9552801; doi:10.3389/fneur.2022.1009113)
Supplement: Supplementary file 1 [file Data_Sheet_1.pdf]

TUDCA-ALS protocol: ethical approvals

National regulatory agencies

Country	National Regulatory Agency	Approval date
Italy	Agenzia Italiana del farmaco (AIFA)	12/12/2018
Germany	Bundesinstitut für Arzneimittel und Medizinprodukte (BfArM)	22/05/2019
United Kingdom	Medicines & Healthcare Products Regulatory Agency (MHRA)	25/04/2019
Belgium	Federal Agency for Medicines and health Products (FAMHP)	11/04/2019
France	Agence Nationale de Sécurité du Médicament (ANSM)	10/05/2019
Ireland	Health Products Regulatory Authority (HPRA)	03/05/2019
Netherlands	Centrale Commissie Mensgebonden Onderzoek (CCMO)	06/06/2019

Ethics committees

Site	Institution	Committee name	Approval date
IT01	IRCCS Istituto Clinico Humanitas	Comitato Etico Indipendente IRCCS Istituto Clinico Humanitas	18/12/2018
IT02	A.O.U. Città della Salute e della Scienza of Torino	Comitato Etico Interaziendale. Segreteria Tecnico-Scientifica AOU Città della Salute e della Scienza di Torino	15/07/2019
IT03	Azienda Ospedaliera Santa Maria di Terni	Comitato Etico delle Aziende Sanitarie della Regione Umbria	22/01/2019
IT04	ASST Grande Ospedale Metropolitano Niguarda Centro Nemo	Comitato Etico "Milano Area C"	22/02/2019
IT05	IRCCS Istituto Auxologico Italiano	Comitato Etico IRCCS Istituto Auxologico Italiano	29/01/2019
IT06	Università della Campania "Luigi Vanvitelli". Azienda Ospedaliera Universitaria	Comitato Etico dell'Università Vanvitelli di Napoli	16/01/2019
DE01	Universität Ulm	Ethikkommission Der Universität Ulm	03/06/2019
DE02	Charité-Universitätsmedizin Berlin	Landesamt für Gesundheit und Soziales (LAGeSo)<br>Geschäftsstelle der Ethik-Kommission des Landes Berlin	03/06/2019
DE03	Alfried Krupp Krankenhaus Rüttenscheid	Ethik-Kommission der Ärztekammer Nordrhein	14/01/2020
DE04	Medizinische Hochschule Hannover	Ethikkommission der Medizinischen Hochschule Hannover	03/06/2019
DE05	Universitätsklinikum Jena	Universitätsklinikum Jena · Ethik-Kommission	03/06/2019
DE06	Universitätsklinikum Carl Gustav Carus Dresden	Ethikkommission an der Technischen Universität Dresden	03/06/2019
UK01	The University of Sheffield – Sheffield Teaching Hospitals – NHS Foundation Trust	East Midlands – Leicester South Research Ethics Committee	20/05/2019

UK02	Lancashire Teaching Hospitals – NHS Foundation Trust	East Midlands – Leicester South Research Ethics Committee	20/05/2019
UK03	Salford Royal – NHS Foundation Trust	East Midlands – Leicester South Research Ethics Committee	20/05/2019
UK04	The Walton Centre – NHS Foundation Trust	East Midlands – Leicester South Research Ethics Committee	20/05/2019
UK05	Plymouth Hospitals – NHS Trust	East Midlands – Leicester South Research Ethics Committee	20/05/2019
UK07	Royal Stoke University Hospital – University Hospitals of North Midlands – NHS Trust<br>Stoke on Trent	"East Midlands – Leicester South Research Ethics Committee<br>The Old Chapel, Royal Standard Place, Nottingham NG1 6FS"	20/11/2019
FR01	Centre Hospitalier Régional Universitaire De Tours. Hôpital Bretonneau	CPP SUD-EST II	26/06/2019
FR02	Hôpital Dupuytren	CPP SUD-EST II	26/06/2019
FR03	Hôpital Gui de Chauliac	CPP SUD-EST II	26/06/2019
FR04	Groupe Hospitalier Pelegrin – Tripode	CPP SUD-EST II	26/06/2019
BE01	Katholieke Universiteit Leuven	Ethics Committee Research UZ / KU Leuven	05/06/2019
NL01	Universitair Medisch Centrum Utrecht	Medical Research Ethics Committee (METC UMC Utrecht)	19/11/2019
IE01	The Provost, Fellows, Foundation Scholars, and the Other Members of Board, of the College of the Holy and Undivided Trinity of Queen Elizabeth Near Dublin	Beaumont Ethics Committee, Beaumont Hospital	23/04/2019
